# Medical School Catalogues

**Published:** 1844-03

**Authors:** 


### MEDICAL SCHOOL CATALOGUES.

We have before us copies of the catalogues of the Medical Schools of Lexington and Louisville, for the sessions just terminated. The Lexington catalogue contains 214 names; that of Louisville 242. The names of 19 resident graduates—*M.D's.* as they are styled—appear in the catalogue of the Lexington school, and but 5 in the catalogue of the Medical Institute of Louisville. Deducting the M. D.'s from each catalogue, the class at Lexington would remain 195, and that at Louisville 237. The Medical Institute, consequently, had more students, by *forty-two*, in attendance on its last course of lectures. It appears, from the catalogue, that the Lexington school, during the first seven years of an eminently prosperous career, attracted 1154 pupils. In the seven years during which the Institute has been in operation, the aggregate of its classes has amounted to 1305, or 151 more than the University. Y.

